# Developing a benchmark for emotional analysis of music

**DOI:** 10.1371/journal.pone.0173392

**Published:** 2017-03-10

**Authors:** Anna Aljanaki, Yi-Hsuan Yang, Mohammad Soleymani

**Affiliations:** 1 Utrecht University, Utrecht, the Netherlands; 2 Swiss Center for Affective Sciences, University of Geneva, Geneva, Switzerland; 3 Academia Sinica, Taipei, Taiwan; Boston Children’s Hospital / Harvard Medical School, UNITED STATES

## Abstract

Music emotion recognition (MER) field rapidly expanded in the last decade. Many new methods and new audio features are developed to improve the performance of MER algorithms. However, it is very difficult to compare the performance of the new methods because of the data representation diversity and scarcity of publicly available data. In this paper, we address these problems by creating a data set and a benchmark for MER. The data set that we release, a MediaEval Database for Emotional Analysis in Music (DEAM), is the largest available data set of dynamic annotations (valence and arousal annotations for 1,802 songs and song excerpts licensed under Creative Commons with 2Hz time resolution). Using DEAM, we organized the ‘Emotion in Music’ task at MediaEval Multimedia Evaluation Campaign from 2013 to 2015. The benchmark attracted, in total, 21 active teams to participate in the challenge. We analyze the results of the benchmark: the winning algorithms and feature-sets. We also describe the design of the benchmark, the evaluation procedures and the data cleaning and transformations that we suggest. The results from the benchmark suggest that the recurrent neural network based approaches combined with large feature-sets work best for dynamic MER.

## Introduction

**Fig 1:**
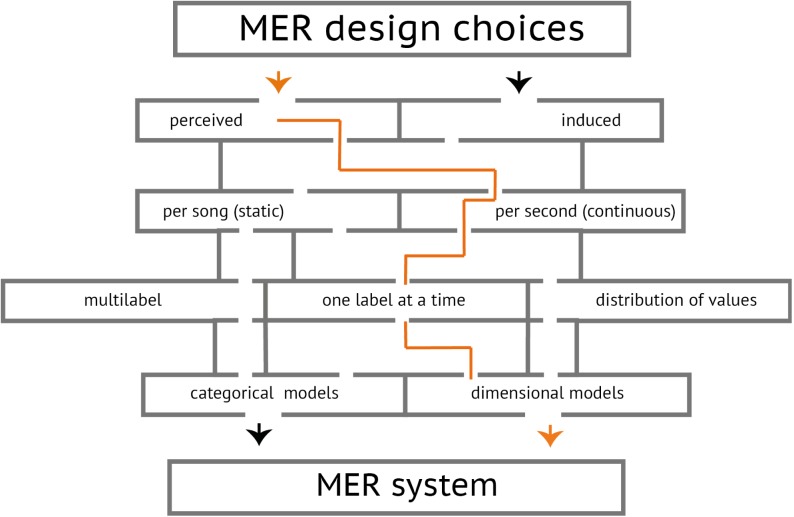
A labyrinth of data representation choices for a MER algorithm. The choices that we made for the benchmark are highlighted in red.

Music emotion recognition (MER) is a young, but fast expanding field, stimulated by the interest from music industry to improve automatic music categorization methods for large-scale online music collections. In [1], an analysis of written music queries from creative professionals showed that 80% of the queries for production music contain emotional terms, making them one of the most salient and important components of exploratory music search. In the last decade, many new MER methods have been proposed (see [2, 3] for reviews). However, methodological differences in data representation result in a choice of different evaluation metrics, which makes the accuracy of the algorithms impossible to compare. Fig 1 shows 14 different data annotation and representation choices in a form of a labyrinth. In addition to these choices, a wide variety of *categorical* and *dimensional* emotional models are used, such as basic emotions [4], valence and arousal model [5–8], Geneva Emotional Music Scales (GEMS) [9, 10], or custom mood clusters [11–13]. Despite differences in data representation, most of the methods are essentially solving the same problem of mapping acoustic features (or lyrics and meta-data based features) to the emotional annotations. A specific learning algorithm can not always be adapted to other representations (though many algorithms, such as SVM or different types of neural networks, are versatile), but audio features are more often transferable. A benchmark can therefore enable a comparison of different methods and feature sets.

Another problem of MER is that due to audio copyright restrictions, the data sets used in various studies are seldom made public and reused in other studies. Annotations are often obtained by crawling the tags from social music websites, such as last.fm or allmusic.com. In this case, the audio is usually copyrighted and can not be redistributed by the researchers. The music that is distributed for free under a license such as Creative Commons, usually is less well-known and has less tags, and therefore needs to be annotated. Annotating with emotional labels is burdensome, because with such a subjective task many annotations are needed for every item.

A fundamental property of music is that it unfolds over time. An emotion expressed in the song may also change over time, though it is always possible to reduce this variety to a single average value. The online music websites, such as moodfuse.com, musicovery.com, allmusic.com, usually represent songs in a mood space by a single label, which is always an approximation of the emotional content of the song. In design of the benchmark, we recognized the time-dependent nature of music by setting out to predict the emotion of the music dynamically (per-second), i.e., the main purpose of the benchmark is to compare *dynamic MER* algorithms, also known as music emotion variation detection (MEVD) algorithms in the literature [2].

In this paper, we describe the design, evaluation metrics and data that we used to benchmark dynamic MER algorithms. MediaEval Database for Emotional Analysis in Music (DEAM) is the combination of the data sets developed in three years (with data transformation and cleaning procedures applied to them), in addition to the manual annotations we received on Amazon Mechanical Turk (MTurk). DEAM contains 1,802 songs (58 full-length songs and 1,744 excerpts of 45 seconds) from a variety of Western popular music genres (rock, pop, electronic, country, Jazz etc). Part of the data was annotated in the lab and part using MTurk platform (http://www.mturk.com). Since the benchmark started in 2013, we have opted for characterizing the emotion of music as numerical values in two dimensions—valence (positive or negative emotions expressed in music) and arousal (energy of the music) (VA) [14, 15], to make it easier to depict the temporal dynamics of emotion variation. We release the full dataset including the averaged and the raw annotations for the benefit of the community (available online at http://cvml.unige.ch/databases/DEAM). Over three years of activity 21 teams participated in the task. We will also systematically evaluate the feature-sets and the algorithms in this paper.

### Background

MER is a young field which has been developing in the last decade. In this section, we will review available benchmarks for MER algorithms.

#### MIREX benchmark and static MER

The only other benchmark that exists for MER methods is the audio mood classification (AMC) task, organized by annual Music Information Retrieval Evaluation eXchange (http://www.music-ir.org/mirex/wiki/) (MIREX) [11]. In this task, 600 audio files are provided to the participants of the task, who have agreed not to distribute the files for commercial purposes. Since 2013, another set of 1,438 segments of 30 seconds clipped from Korean pop songs has been added to MIREX. The benchmark uses five discrete emotion clusters, derived from cluster analysis of online tags, instead of more widely accepted dimensional or categorical models of emotion. Emotional model used in AMC has been the topic of debate since it is not based on psychological research. There is also semantic or acoustic overlap between clusters [16]. Furthermore, the dataset only applies a singular static rating per audio clip (i.e. it deals with the *static MER* problem), which does not take into account the temporally dynamic nature of music.

#### Dynamic MER methods

Since the late 1980s, time-varying responses to music were measured using Continuous Response Digital Interface [17]. Usually, only one dimension (such as tension, musical intensity or emotionality) was measured. Schubert proposed to use two-dimensional interface (valence–arousal plane) to annotate music with emotion continuously [18]. This approach was adopted by MER researchers as well.

The first study that models musical emotion unfolding over time with musical features (loudness, tempo, melodic contour, texture, and spectral centroid) was conducted by Schubert in 2004 [19]. The model, using a linear regression, could explain from 33% to 73% of variation in emotion. In 2006, Korhonen *et al.* [20] suggested a method to model musical emotion as a function of musical features using system identification techniques. Korhonen *et al.* used the low-level spectral features extracted using Marsyas software (http://marsyas.info), and perceptual features extracted with PsySound software [21]. The system reached a performance of 0.22 for valence and 0.78 for arousal in terms of the coefficient of determination (*R*^2^). In 2010, Schmidt *et al.* [22] used Kalman filtering to predict per-second changes in the distribution of emotion over time on 15 second music excerpts. In 2011, Schmidt and Kim suggested using a new method—Conditional Random Fields—to model continuous emotion with a resolution of 11 × 11 in valence–arousal space [23]. A very small feature-set was used—MFCCs, spectral contrast and timbre—and the system reached performance of 0.173 in terms of Earth Mover’s Distance (between the true 11 × 11 2D histogram of valence–arousal values and predicted one). Panda *et al.* [24] used Support Vector Machines and features extracted with Marsyas and MIRToolbox to track music over quadrants of valence–arousal space. Imbrasaite *et al.* [25] combined Continuous Conditional Random Fields with a relative representation of features. Later, Imbrasaite *et al.* [26] showed that using Continuous Conditional Neural Fields offers improvement over the previous approach. Wang *et al.* [27] represented the ambiguity of emotion through a Gaussian distribution and tracked the emotion variation over time using a mapping between music emotion space and low-level acoustic feature space through a set of latent feature classes. Markov *et al.* [28] used Gaussian Processes for dynamic MER. The bidirectional Long Short-Term Memory Recurrent Neural Networks were first applied to continuous emotion recognition not in the domain of music, but in the domain of multimodal human emotion detection from speech, facial expression and shoulder gesture [29].

Most of the algorithms mentioned in this section were employed in the benchmark: Support Vector Regression, linear regression, Kalman filtering, Gaussian Processes, Conditional Random Fields, Continuous Conditional Neural Fields and Long Short-Term Memory Recurrent Neural Networks, giving us an opportunity to qualitatively compare their performance in the benchmark.

#### Datasets for dynamic MER

Most of the studies reviewed above did not release public data. The only exception is the MoodSwings dataset [30], developed by Schmidt *et al.*, which comprises 240 segments of US pop songs (each 15-second long) with per-second VA annotations, collected through MTurk. After an automatic verification step that removed unreliable annotations, each clip in this dataset was annotated by 7 to 23 subjects.

A similar task from a different domain is continuous emotion recognition from human behavior. Audiovisual emotion challenge (AVEC) [31–35] is a challenge that has been running since 2011 and is addressing the problem of continuous emotion recognition. Since 2011, they used SEMAINE [36] and RECOLA [37] databases which include human behavior with continuous emotion labels. There are also public datasets with static per song music emotion annotations. The DEAP dataset [38] has the ratings on valence, arousal and dominance for 120 clips of one-minute music video clips of Western pop music. Each clip was annotated by 14–16 listeners (50% female), who were asked to rate the felt valence, arousal and dominance on a 9-point scale for each clip. The AMG1608 dataset [39] contains the VA ratings for 1,608 Western music in different genres, also annotated through MTurk.

## Music database

Our data set consists of royalty-free (Creative Commons license enables us to redistribute the content) music from several sources: freemusicarchive.org (FMA), jamendo.com, and the medleyDB dataset [40]. There are 1,744 clips of 45 seconds from FMA and 58 full length songs, half of which come from medleyDB and another half from Jamendo.

The music from the FMA was in *rock, pop, soul, blues, electronic, classical, hip-hop, international, experimental, folk, jazz, country* and *pop* genres. The music from the MedleyDB dataset in addition had music in *world* and *rap* genres, and the music from Jamendo also had *reggae* music. For 2014 and 2015 data set, we manually checked the music and excluded the files with bad recording quality or those containing speech or noise instead of music. For each artist, we selected no more than 5 songs to be included in the dataset. For medleyDB and Jamendo full-length songs, we selected songs which had emotional variation in them, using an existing dynamic MER algorithm for filtering and manual final selection [41].

## Annotations

**Fig 2:**
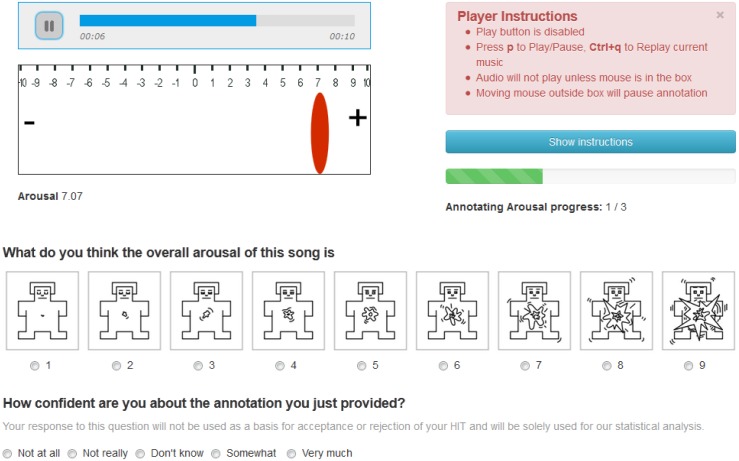
Annotation interface for both continuous (upper-left corner) and static per song (middle; using the self-assessment manikins [43]) ratings of arousal.

Getting high quality data is a crucial step for a highly subjective task. To collect annotations, we have turned to crowdsourcing using MTurk, which was successfully used by others to label large libraries [30, 39]. We developed a procedure to filter out poor quality workers, following current state-of-the-art crowdsourcing approaches [42]. The workers passed a test to demonstrate a thorough understanding of the task, and an ability to produce good quality work. The test contained several automatically scored multiple choice questions, and several free-form questions and assignments, which were evaluated manually if the automatically scored part was passed correctly. In years 2013 and 2014, each excerpt was annotated by a minimum of 10 workers. In 2015, each song was annotated by five workers, three of which were recruited among the most successful workers from previous years, and two were working in the lab. To ensure a high-quality outcome, we first discussed and set a fair compensation for such a demanding task (about $8 per hour) on a MTurk workers’ forum (http://www.mturkgrind.com/). We then double-checked the agreement between the annotators in each batch before assigning an increasing qualification score which permitted workers to work on the next batches. The dynamic annotations were collected using a web-interface on a scale from −10 to 10, where the Mechanical Turk workers could dynamically annotate the songs on valence and arousal dimensions separately while the song was being played. The static annotations were made on nine-point scale on valence and arousal for the whole 45 seconds excerpts after the dynamic annotations. Fig 2 shows the interface used for annotation.

As summarized in Table 1, in addition to the audio features, we also provide meta-data covering the genre labels obtained from FMA, medleyDB and Jamendo, folksonomy tags crawled from last.fm, and meta-data about the annotators.

**Table 1:** The data overview of DEAM.

Year	Number of songs	Source	Meta-data about music	Meta-data about annotators
2013	1000 (744 unique)	MTurk	Genre	Time of the day, mood
2014	1000	MTurk	Genre	Confidence in rating, familiarity of music, liking of music, free emotion label, Big Five personality, preferred genre
2015	58	MTurk/Lab	Genre	Liking, free emotion label

### Annotation consistency

We will evaluate annotation consistency using two measures: Cronbach’s *α* on the sequences of annotations for each of the songs, and coefficient of determination of a Generalized Additive Model that generalizes song’s annotations across annotators.

We resample the annotations to 2Hz, and normalize the annotations for each song by

$$\begin{array}{c} a_{j,i}=a_{j,i}+\left(\bar{A_{j}}-\bar{A}\right)\;, \end{array}$$ (1)

where *a*_*j*,*i*_ is an annotation by annotator *j* at timestamp *i*, $\bar{A_{j}}$ is the mean of the annotations by annotator *j*, and $\bar{A}$ is a mean of all annotations for this song by all annotators (global mean).

Cronbach’s *α* is used to estimate the degree to which a set of items measures a single unidimensional latent construct. This measure should theoretically range between 0 and 1, but in practice can be negative when inter-item correlations are negative. There is no lower bound on negative values of this measure. Only positive values are informative and accurately report degree of agreement. Therefore, we clip the negative tail by assigning the value of 0. Table 2 shows the averaged Cronbach’s *α* for each year’s annotations. To test whether annotation consistency improved with a change of experimental design, we will compare the three groups. Groups’ sample sizes and variances are different, therefore we will use a non-parametric test based on ranks. Kruskal-Wallis test (one way ANOVA on ranks) shows that there are significant differences between groups for arousal (*χ*^2^(2) = 81.24, *p*-value = 2.2 × 10^−16^) and Dunnett-Tukey-Kramer test shows that the differences are significant between all three years on a 1% significance level. For valence, the differences exist (*χ*^2^(2) = 57.91, *p*-value = 2.6 × 10^−13^), but only annotations from 2015 are significantly different from other groups.

**Table 2:** Annotation consistency. Cronbach’s *α* and generalized additive mixed models (GAM)’s coefficient of determination (mean and standard deviation) per year.

Year	2013	2014	2015
Total length	9h 18min	12h 30min	3h 46min
Cronbach’s *α* for arousal	.28 ± 0.28	.31 ± 0.30	.66 ± 0.26
GAM’s *R*^2^ for arousal	.13 ± 0.10	.14 ± 0.11	.44 ± 0.19
Cronbach’s *α* for valence	.28 ± 0.29	.20 ± 0.24	.51 ± 0.35
GAM’s *R*^2^ for valence	.13 ± 0.10	.10 ± 0.08	.37 ± 0.21

Cronbach’s *α* test has some deficiencies, such as being sensitive to the number of items on the test (greater number of items in the test can artificially inflate the value of alpha). Therefore, we conduct an additional consistency test with generalized additive mixed models (GAMs) [44]. A GAM is a generalized (i.e., allowing non-normal error distributions of the response variable) linear model with a linear predictor involving a sum of smooth functions of covariates. The model is defined as follows:

$$\begin{array}{c} g\left(\mu \right)=\beta_{0}+f_{1}\left(x_{1}\right)+f_{2}\left(x_{2}\right)+...+f_{n}\left(x_{n}\right)\;, \end{array}$$ (2)

where *g* is a link function (a function defining a relationship between the linear predictor and the mean of the dependent variable); *μ* = *E*(*Y*), where *Y* is a dependent variable; and *f*_*i*_(*x*_*i*_) are non-parametric smooth functions, estimated, e.g. via scatterplot smoothing techniques, or can also be parametric functions or factors.

GAMs are suitable for modeling continuous annotations of emotion, because these annotations are usually non-linear in nature and do not have abrupt changes, making it possible to model them using smooth functions. KcKeown and Sneddon [44] described how GAMs and their mixed model extension can be used to model continuous emotion annotations and make inferences concerning linear differences between groups. In this paper, we only use GAMs to assess the effect size of shared perceived emotion. This is done by building a model for each song and calculating the *R*^2^ of the model.

**Fig 3:**
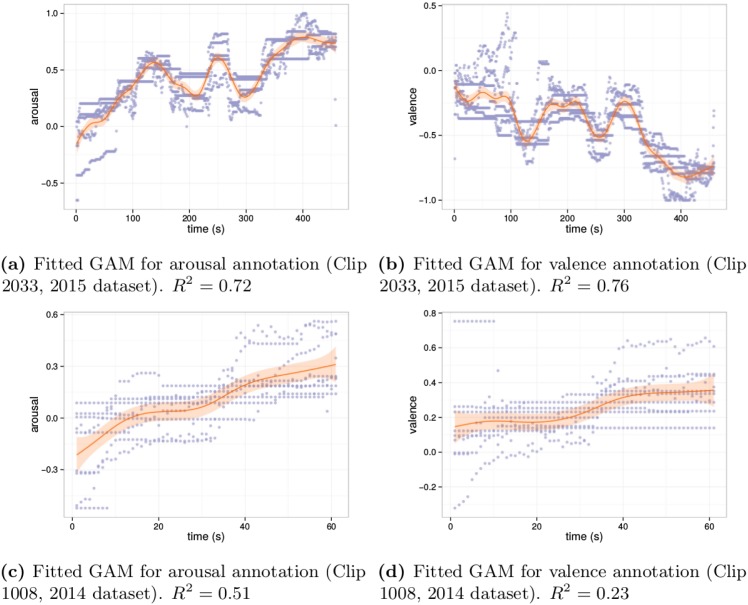
Fitted GAMs for the arousal and valence annotations of two songs.

There is only one smooth component in the model—time. We use penalized cubic regression splines with basis dimension of 20 and identity link function. The results are shown in Table 2. Fig 3 shows scatter-plots of the manual annotations and the fitted GAMS for 2 songs picked from 2015 dataset.

There are significant differences between groups for arousal according to Kruskal-Wallis test (*χ*^2^(2) = 121.03, *p*-value = 2.2 × 10^−16^) and Dunnett-Tukey-Kramer test shows that the differences are significant between 2015 and other groups on a 1% significance level. For valence, the outcome is the same: differences exist (*χ*^2^(2) = 134.37, *p*-value = 2.2 × 10^−16^), and only year’s 2015 annotations are significantly different from other groups.

According to both consistency measures, in 2015 we could achieve better consistency, which can be attributed to employing lab workers, choosing complete songs over excerpts and introducing preliminary listening.

#### Influence of music familiarity, liking and other factors on annotations

The Creative Commons music that we selected was largely unfamiliar to participants (only in 1% of the listening sessions the participant reported having heard the piece before). Hence, there was not enough data to derive any patterns regarding the familiarity of the music.

**Fig 4:**
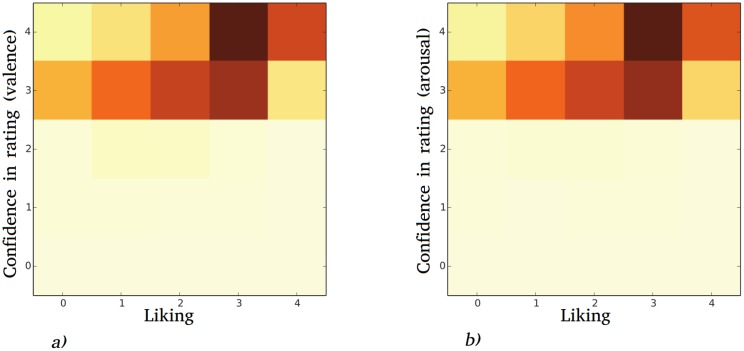
Liking of the music and confidence in rating for a) valence, Spearman’s *ρ* = 0.37, *p*-value = 2.2 × 10^−16^ b) arousal, Spearman’s *ρ* = 0.29, *p*-value = 2.2 × 10^−16^.

We found that liking influenced self evaluation of confidence in rating. Fig 4 shows the 2D histogram for self-reported confidence in rating and liking the music. The confidence in rating is on average very high (the workers never reported being very uncertain), which is, probably, caused by the fact that the data was collected from paid workers who did not want to be suspected of incompetence. Liking the music influenced perceived self-reported confidence. A similar effect was found in [45], when there was a positive dependency between liking the music and annotation consistency. We could not find any effect of averaged music liking on actual rating consistency as measured by correlation of a rating with other workers, or Cronbach’s *α* of a song.

#### Convergence of annotations

It is a known issue that the annotators need some initial orientation time (IOT), before their continuous annotations become meaningful and reliable. In [46], median IOT was found to be 8 seconds for valence and 12 seconds for arousal. Also, afterglow effects—large outliers in spread of scores just after the end of a piece—were identified. In [47], participants required on average 8.31 seconds to initiate giving emotional judgements on music on a two-dimensional plane. The length of delay was influenced by familiarity, genre and tempo of music.

**Fig 5:**
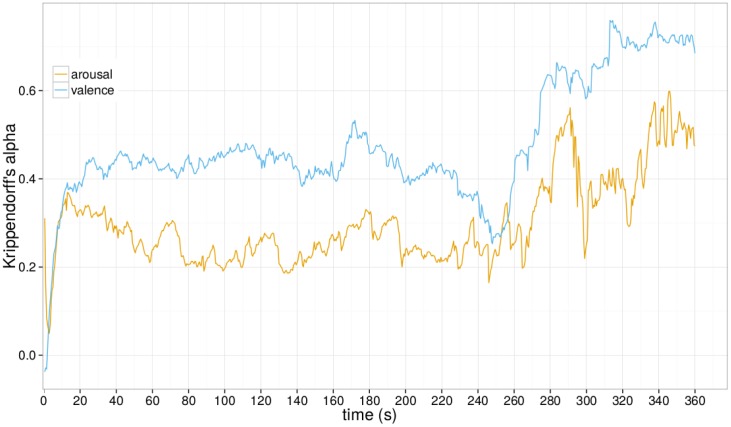
Krippendorff’s *α* of dynamic annotations in 2015, averaged over all dynamic samples.

To measure the IOT of the annotators in the beginning of the song, we calculate the average Krippendorff’s *α* for every sample of the corresponding second for the whole dataset of 2015. The songs in the dataset had different length. Fig 5 shows that the annotations start to converge around the 13^th^ second. A similar result was obtained in 2013^th^ annotations [48]. So, despite preliminary listening stage, the reaction time did not diminish.

We remove the first 15 seconds of the annotation from the benchmark data.

## Benchmark history and design

The benchmark for music emotion recognition algorithms, described in this article, was organized in years 2013-2015 inside the MediaEval Benchmarking Initiative for Multimedia Evaluation (http://www.multimediaeval.org). MediaEval is a community-driven benchmark dedicated to evaluating algorithms for multimedia access and retrieval, that is organized annually since year 2008 (as VideoCLEF, in years 2008 and 2009). The list of tasks offered at the benchmark is renewed every year based on interest and feedback from the multimedia retrieval community. Alongside Emotion in Music task, 10-11 other tasks related to speech, music, image and video processing were held at MediaEval in years 2013-2015. We followed MediaEval benchmarking tradition, by developing a separate development and evaluation-set for each year.

### Task definition 2013

In year 2013, the task was first proposed and organized inside MediaEval framework by Mohammad Soleymani, Yi-Hsuan Yang and Erik Schmidt [49]. The task consisted of two subtasks: *dynamic* and *static* emotion characterization. In dynamic emotion characterization, the participating algorithms predicted emotion (valence and arousal) of the music dynamically per-second. In the static task, the valence and arousal of the complete music clip (45 seconds) were predicted. The training data set consisted of 700 excerpts of 45 seconds, which were labelled both with dynamic annotation (1Hz) and static annotation, where static was not derived from dynamic, but was given separately. 300 clips were left out for the evaluation-set. The music came from Free Music Archive. Later, duplicates (excerpts sampled from the same song) were discovered and removed from this data, leaving 744 clips out of 1000.

### Task definition 2014

In 2014, the static emotion characterization task was removed and a new subtask—*feature design*—was added instead [50]. In the feature design task, new features, which have not been developed before, were proposed and applied to valence and arousal prediction task. The feature design task was not popular and only one team submitted to that task [51]. The training set consisted of 744 clips from previous year and 1000 new clips, all from Free Music Archive, served as the evaluation-set. The time resolution for the dynamic task was changed to 2Hz.

### Task definition 2015

In 2015, the feature design subtask was removed, leaving only dynamic emotion characterization task. The training set consisted of 431 clips, which were selected out of 1,744 clips from previous years based on consistency metrics. The data cleaning procedure is described in [41]. The evaluation-set consisted of 58 full length songs, one half from the medleyDB dataset [40] of royalty-free multitrack recordings and another half from the jamendo.com music website, which provides music under Creative Commons license. The songs were ≈4 minutes (234 ± 107 s) long on average. The time resolution for the annotations was 2Hz. The participants had to submit:

- Features that the participants used in their approach. The features were used to train a baseline regression method (linear regression) to estimate dynamic affect. Any features automatically extracted from the audio or the meta-data provided by the organizers were allowed.
- Predictions using baseline features.
- Predictions using any combination of the features and machine learning methods of their choice.

### Evaluation metrics

We used two evaluation metrics to compare the performance of different methods: Pearson’s correlation coefficient between the ground truth and predicted values for each song, averaged across songs, and root mean square error (RMSE), averaged the same way. In years 2013 and 2014, we used correlation coefficient as the main metric and RMSE as an auxiliary metric to break the ties. The tie is a situation, when the difference between two methods adjacent in the ranking is not significant based on the one sided Wilcoxon test (*p* < 0.05). In 2015, we used RMSE as our primary metric. RMSE metric measures how far is the prediction of the emotion from the true emotion of the song, and correlation measures whether the direction of change is guessed correctly. However, in case of dynamic emotions, the trend shape of the traces are also important. In this paper, we will also report concordance correlation coefficient (CCC) *ρ*_*c*_ as an evaluation metric. This metric was suggested by Lin [52] in 1989 and is defined as follows:

$$\begin{array}{c} \rho_{c}=\frac{2s_{xy}}{s_{x}^{2}+s_{y}^{2}+\left(\bar{x}+\bar{y}\right)}, \end{array}$$ (3)

where *x* and *y* are the vectors of numbers to compare, $s_{x}^{2}$ is the variance of x, *s*_*xy*_ is the covariance of *x* and *y*, and $\bar{x}$ is the mean of vector *x*. CCC has recently been promoted as the metric of choice for continuous emotion recognition [35].

### Baseline features

In every year, baseline features extracted from the audio were offered to the participants along with the audio files. In the majority of cases, these features were used by the participants in their submissions. In year 2013, the features were MFCCs, octave- based spectral contrast, spectral features (centroid, flux, rolloff, flatness), chromagram, and timbre, pitch, and loudness features from the Echonest 7 API. In year 2014, we released the features extracted with openSMILE toolbox [53] as described in [54]. In year 2015, we extracted a smaller set of features with openSMILE. We obtained 260 low-level features (mean and standard deviation of 65 low-level acoustic descriptors, and their first-order derivatives) from non-overlapping segments of 500ms, with the frame size of 60ms with a 10ms step.

No feature selection was applied when building baseline linear regression models from baseline features. In year 2014, as an exception, different features were used to build a baseline model (spectral flux, harmonic change detection function, loudness, roughness and zero crossing rate).

## Analysis of proposed methods

In this section, we will analyze the best systems suggested over the three years of benchmark. In the last edition of the benchmark (2015), we asked the participants to provide their feature-sets, and to run their algorithms on the baseline feature-set. In this way, we can conduct a systematic evaluation of the algorithms and feature-sets separately.

### Task participation

Three teams participated in the task in year 2014 and the results were analyzed in [48]. In 2014, there were six teams and in 2015, twelve teams. Every team wrote a working notes paper which is available in the proceedings on the corresponding year MediaEval workshop. The last edition of the benchmark had most participating teams, and most of the algorithms from the previous years featured in the last edition. In this paper, we will mostly analyze the results of the benchmark held in 2015.

### Performance in a challenge over years

Tables 3–5 show the results of the benchmark in year 2013, 2014 and 2015. The results are sorted by RMSE of arousal ascending (best solutions on top). Column “Method” shows the abbreviation of the machine learning algorithm used by a particular team, and a working notes paper that was published in the proceedings, where the details of the approach are explained. All the methods beat the baseline, shown on the bottom row. The baseline method is a multi-linear regression with openSMILE features.

**Table 3:** Performance of the algorithms for arousal and valence in year 2013. **BLSTM-RNN**—Bi-directional Long-Short Term Memory Recurrent Neural Networks. **GPR**—Gaussian Processes Regression. **SVR**—Support Vector Regression.

Method	Arousal		Valence	
	RMSE	*ρ*	RMSE	*ρ*
**BLSTM-RNN** [54]	.08 ± .05	.31 ± .37	.08 ± .04	.19 ± .43
**GPR** [28]	.10 ± .05	.11 ± .36	.09 ± .05	.06 ± .28
**SVR** [57]	.10 ± .06	.14 ± .28	.12 ± .07	−.01 ± .27
**Baseline**	.25 ± .11	.16 ± .36	.23 ± .10	.06 ± .30

**Table 4:** Performance of the algorithms for arousal and valence in year 2014. **KF**—Kalman Filter. **LSTM**—Long-Short Term Memory Recurrent Neural Network. **CCRF**—Continuous Conditional Random Fields. **CCNF**—Continuous Conditional Neural Fields. **MR**—Multi-level regression. **PLSR**—Partial Least Squares Regression.

Method	Arousal		Valence	
	RMSE	*ρ*	RMSE	*ρ*
**KF** [56]	.08 ± .05	.21 ± .57	.14 ± .07	.17 ± .5
**LSTM** [55]	.10 ± .05	.35 ± .45	.08 ± .05	.20 ± .49
**CCRF** [58]	.12 ± .05	.23 ± .56	.09 ± .05	.12 ± .55
**CCNF** [26]	.12 ± .07	.18 ± .60	.10 ± .06	.07 ± .29
**MR** [59]	.12 ± .05	.17 ± .41	.09 ± .05	.10 ± .37
**PLSR** [51]	.13 ± .07	.28 ± .50	.10 ± .06	.15 ± .5
**Baseline**	.14 ± .06	.18 ± .36	.10 ± .06	.11 ± .34

**Table 5:** Performance of the algorithms for arousal and valence in 2015. **BLSTM-ELM**—BLSTM-based multi-scale regression fusion with Extreme Learning Machine. **AE-HE-BLSTM**—BLSTM + features created through deep learning. **LS**—Linear regression + Smoothing. **LSB**—Least Squares Boosting + Smoothing. **SVR + CCRF**—SVR + Continuous Conditional Random Fields.

Method	Arousal		Valence	
	RMSE	*ρ*	RMSE	*ρ*
**BLSTM-RNN** [60]	.12 ± 0.06	.66 ± 0.25	.17 ± 0.09	.12 ± 0.54
**BLSTM-ELM** [60]	.12 ± 0.05	.63 ± 0.27	.15 ± 0.08	.15 ± 0.47
**LSTM-RNN** [61]	.12 ± 0.06	.61 ± 0.28	.19 ± 0.10	.03 ± 0.50
**LSTM-RNN** [61]	.12 ± 0.06	.60 ± 0.29	.19 ± 0.10	.02 ± 0.49
**LS** [62]	.12 ± 0.05	.65 ± 0.22	.17 ± 0.09	.01 ± 0.50
**LSB** [62]	.12 ± 0.05	.59 ± 0.23	.17 ± 0.09	.05 ± 0.43
**SVR** [63]	.12 ± 0.05	.56 ± 0.27	.19 ± 0.10	−0.02 ± 0.45
**SVR + CCRF** [64]	.12 ± 0.05	.54 ± 0.27	.17 ± 0.09	.02 ± 0.43
**AE-HE-BLSTM** [60]	.12 ± 0.06	.52 ± 0.37	.17 ± 0.09	.02 ± 0.51
**LSTM-RNN** [61]	.12 ± 0.06	.61 ± 0.25	.19 ± 0.10	.00 ± 0.50
**Baseline**	.14 ± 0.06	.37 ± 0.26	.18 ± 0.09	−0.01 ± 0.38

In year 2013 and 2015, LSTM-RNN based solutions were the best both for arousal and valence, in year 2014 LSTM-based solution was second best for arousal, but best for valence.

In year 2013, all the teams used different feature-sets. The results are analyzed in detail in [48].

In year 2014, solutions [26] and [55] used openSMILE feature-sets. The rest of the teams used other features. The combination that produced the best result for arousal (but worse than baseline result for valence) [56], was a combination of a Kalman filter and low-level features: MFCCs, zero-crossing rate, spectral flux, centroid, rolloff, and spectral crest factor.

Table 5 shows only 10 best solutions for 2015. Each of the 12 teams submitted 3 runs, which creates more than 30 different solutions, some of which were on par with the baseline. All of the solutions listed use the baseline openSMILE feature-set, but it is usually transformed, or new features are added.

### Evaluation of the machine learning algorithms

In this section, we describe an evaluation of the algorithms on the same feature-set (the baseline features of year 2015).

Table 6 shows the evaluation of the algorithms participating in 2015 challenge on this feature-set. 10 best approaches are reported. The performance in terms of RMSE for arousal is the same for all the solutions (though correlation coefficient is different), indicating that the algorithms might have reached some sort of ceiling in performance with this combination of annotations and features.

**Table 6:** Performance of the different algorithms for arousal and valence, using the baseline feature-set. **Combo**—An unweighted combination of LS, LSB and Boosted ensemble of single feature filters.

Method	Arousal			Valence		
	RMSE	*ρ*	*ρ*_*c*_	RMSE	*ρ*	*ρ*_*c*_
**BLSTM-RNN** [60]	.12 ± .06	.66 ± .25	.30 ± .24	.15 ± .08	.15 ± .47	.06 ± .17
**BLSTM-ELM** [60]	.12 ± .05	.63 ± .27	.25 ± .22	.15 ± .08	.15 ± .47	.06 ± .17
**LR + S** [62]	.12 ± .05	.65 ± .22	.32 ± .23	.17 ± .09	.01 ± .50	.01 ± .19
**LSB** [62]	.12 ± .05	.59 ± .23	.30 ± .24	.17 ± .09	.05 ± .43	.01 ± .18
**LSTM-RNN** [61]	.12 ± .06	.61 ± .25	.31 ± .26	.19 ± .10	.00 ± .50	.01 ± .20
**Combo** [62]	.12 ± .05	.64 ± .23	.28 ± .22	.17 ± .09	.00 ± .48	.01 ± .19
**SVR** [63]	.12 ± .05	.56 ± .27	.31 ± .25	.19 ± .10	−0.02 ± .45	.00 ± .18
**SVR + CCRF** [64]	.12 ± .05	.52 ± .30	.22 ± .22	.17 ± .10	.00 ± .43	.00 ± .13
**LSTM-RNN** [65]	.12 ± .06	.59 ± .24	.30 ± .23	.18 ± .09	.03 ± .48	.00 ± .20
**SVR** [60]	.12 ± .07	.56 ± .24	.08 ± .08	.15 ± .09	.01 ± .40	.00 ± .04
**Baseline - FNN**	.12 ± .08	.45 ± .22	.25 ± .20	.14 ± .13	.00 ± .41	.04 ± .16

The algorithms are sorted by their performance according to RMSE on arousal ascending (RMSE increases and performance decreases). The algorithms show very good performance on arousal and completely unsatisfactory performance on valence. It is a known issue, that valence is much more difficult to model than arousal, but not to the extent that we observe.

**Fig 6:**
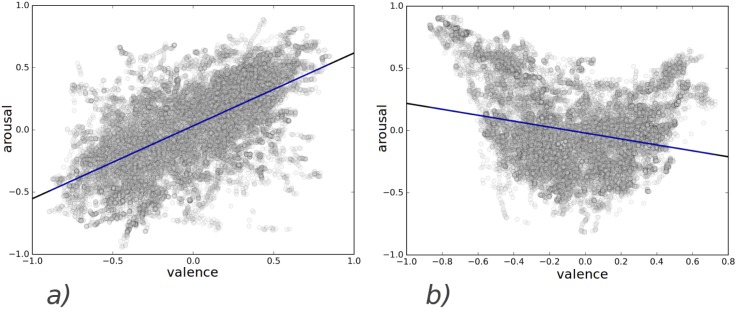
Distribution of the labels on arousal-valence plane for a) development-set b) evaluation-set.

In 2013 and 2014, valence and arousal annotations were highly correlated whereas in 2015, they were not. We hypothesize that due to the high correlation the algorithms did not train to recognize valence-specific cues and could not perform well on the evaluation-set. Fig 6 shows the scatter plots of the annotations along with regression lines.

Almost all the solutions listed in Table 6 are either based on LSTM-RNN networks or SVR. Exception are solutions suggested by team SAILUSC [62], which are based on linear regression with smoothing, or least squares boosting. LSTM-RNN networks are capable of incorporating local context in their predictions. A smoothing step also incorporates the context, though it can not learn the dependencies in time-series. We also provide a baseline Feed-Forward Neural Netword (FNN)—dropout-regularized neural net with three hidden layers. Without any smoothing step, the feed-forward neural net demonstrates worse performance in terms of Pearson’s correlation coefficient. With median-filter smoothing applied to results, the correlation coefficient for arousal is similar to the rest of the approaches (0.57 ± 0.24).

### Evaluation of the feature-sets

In this section, we will analyze the features proposed by the teams in 2015 through building a system using the same machine learning algorithm, but different feature-sets. We chose the best performing algorithm of previous years—LSTM-RNN.

We constructed a network with three hidden layers with 250, 150 and 50 nodes, similar to the architecture used by ICL team. We used the number of memory blocks in each hidden layer, the learning rate (LR), and the standard deviation of the Gaussian noise applied to the input activations, which were optimized for our data by the ICL team [61]. Every layer was pretrained (in a supervised way) before the next layer was added and the network was trained again. We used 20-fold cross-validation for evaluating results.

#### Proposed features

A variety of software for audio signal processing and feature extraction was used by participants: Marsyas, MIRToolbox for Matlab, PsySound, openSMILE, Essentia, jAudio. Mostly, participants used the features that are known to be important for emotion recognition, such as MFCCs, tempo, loudness, low level spectral features related to timbre. Few novel features were proposed. Kumar *et al.* [51] proposed three new types of features: compressibility features, which describe how much the audio can be compressed, median spectral band energy, which describes the spectral bandwidth of the audio. The compressibility of audio was strongly positively correlated with static arousal ratings (Pearson’s *r* = 0.656). Cai *et al.* [66] proposed edge orientation histograms on mel-frequency spectrogram.

#### Results on development and evaluation-set cross-validation

Table 7 shows the evaluation of the feature-sets on valence, ordered by Concordance Correlation Coefficient of the results on evaluation-set, descending. The best performing feature-set for valence (by JUNLP team) is a baseline feature-set with feature selection applied to it to find the features optimized for valence recognition. The second best feature-set, suggested by PKUAIPL team, consisted of the baseline feature-set with an addition of three types of features: MFCCs and Δ MFCCs, edge-orientation histograms and standard low-level spectral features. In addition, team PKUAIPL applied auto-regressive and moving average filters to the features to account for the temporal changes in music, and added the output as new features to the feature vector. Team HKPOLYU suggested a supervised transformation on the baseline feature-set (valence–arousal similarity preserving embedding). This transformation maps high-dimensional feature vectors to a lower-dimensional space so that for similar songs (in terms of valence or arousal) the feature vectors are also closer in this low-dimensional space.

**Table 7:** Performance of the different feature-sets on valence, development and evaluation-sets of 2015, 20 fold cross-validation.

Method	Development-set			Evaluation-set		
	RMSE	*ρ*	*ρ*_*c*_	RMSE	*ρ*	*ρ*_*c*_
**JUNLP (2)** [67]	.26 ± .15	.22 ± .51	.09 ± .24	.27 ± .13	.19 ± .35	.08 ± .15
**PKUAIPL** [64]	.22 ± .13	.33 ± .50	.16 ± .27	.27 ± .14	.16 ± .35	.07 ± .20
**HKPOLYU** [63]	.21 ± .13	.41 ± .53	.20 ± .28	.28 ± .14	.19 ± .36	.06 ± .17
**JUNLP (3)** [67]	.26 ± .15	.23 ± .53	.09 ± .24	.28 ± .13	.17 ± .33	.06 ± .14
**UNIZA (1)** [68]	.22 ± .14	.32 ± .50	.16 ± .27	.29 ± .14	.14 ± .37	.06 ± .14
**ICL** [61]	.22 ± .13	.30 ± .50	.15 ± .27	.30 ± .14	.12 ± .40	.06 ± .16
**JUNLP (1)** [67]	.22 ± .14	.32 ± .50	.15 ± .27	.28 ± .13	.12 ± .39	.05 ± .15
**UNIZA (2)** [68]	.23 ± .14	.31 ± .49	.15 ± .26	.29 ± .16	.09 ± .40	.05 ± .17
**IRIT-SAMOVA** [65]	.23 ± .14	.33 ± .50	.16 ± .27	.29 ± .15	.08 ± .41	.05 ± .16
**MIRUtrecht** [69]	.24 ± .15	.30 ± .49	.13 ± .23	.29 ± .14	.11 ± .43	.04 ± .15

Table 8 shows the evaluation of the feature-sets on arousal, ordered by Concordance Correlation Coefficient of the results of development-set, descending. Teams HKPOLYU, THU-HCSIL and IRIT-SAMOVA suggested the best features for arousal. The features by the team HKPOLYU were already described above. Team THU-HCSIL applied Deep Belief Networks to a set of features extracted with openSMILE and MIRToolbox, in order to learn the higher representation for each group features independently, which were then fused by a special autoencoder with a modified cost function considering sparse and heterogeneous entropy, to produce the final features at a rate of 2Hz for the succeeding regression. Team IRIT-SAMOVA could achieve a very good performance with a very simple feature-set consisting of 6 measurements on bands of a Bark scale for spectral valley, and spectral flatness on ERB and Bark scale, for a total of only 8 features. Spectral flatness provides a way to quantify how noise-like a sound is. Spectral valley is a feature derived from the so-called spectral contrast feature, which represents the relative spectral distribution.

**Table 8:** Performance of the different feature-sets on arousal, development and evaluation-sets of 2015, 20 fold cross-validation.

Method	Development-set			Evaluation-set		
	RMSE	*ρ*	*ρ*_*c*_	RMSE	*ρ*	*ρ*_*c*_
**HKPOLYU** [63]	.20 ± .12	.48 ± .47	.23 ± .28	.22 ± .12	.39 ± .41	.24 ± .26
**THU-HCSIL** [60]	.21 ± .13	.46 ± .42	.22 ± .26	.27 ± .12	.33 ± .40	.16 ± .22
**IRIT-SAMOVA (3)** [65]	.21 ± .13	.49 ± .43	.21 ± .27	.24 ± .13	.52 ± .37	.25 ± .25
**IRIT-SAMOVA** [65]	.21 ± .12	.45 ± .44	.21 ± .27	.24 ± .12	.43 ± .30	.22 ± .22
**JUNLP (1)** [67]	.21 ± .12	.45 ± .43	.19 ± .26	.24 ± .12	.52 ± .31	.26 ± .24
**UNIZA (2)** [68]	.22 ± .12	.41 ± .44	.19 ± .25	.24 ± .12	.48 ± .32	.26 ± .24
**PKU-AIPL** [64]	.21 ± .12	.40 ± .44	.19 ± .26	.23 ± .10	.52 ± .30	.32 ± .27
**UNIZA (1)** [68]	.22 ± .12	.40 ± .44	.19 ± .25	.25 ± .13	.49 ± .30	.27 ± .23
**JKU-Tinnitus (2)** [70]	.22 ± .13	.39 ± .45	.19 ± .26	.30 ± .14	.06 ± .38	.04 ± .17
**JKU-Tinnitus (1)** [70]	.22 ± .12	.38 ± .43	.19 ± .26	.29 ± .14	.09 ± .39	.05 ± .15

## Discussions and perspectives

During the three years of organizing the ‘Emotion in Music’ task at MediaEval, changes were introduced to the data collection routine, which led to the improvement of the quality of the annotations. In the first two years of the benchmark, the size of the segment was chosen in such a way that both static and dynamic ratings were possible. This resulted in a compromise, which led to selecting the window of 45 seconds, which appears to be too short to capture a lot of emotional variation, and too long to make estimating the static emotion unambiguous. In 2015, we opted for full-length songs. In combination with preliminary listening and more careful selection of workers, the quality of the annotations was improved. However, full-length songs might also not be the optimal solution because the annotation procedure is very demanding and requires a lot of concentration, and there is a danger that full-length song annotation stretches the limits of what human annotators are capable of. This question requires more investigation. Also, in 2015 we employed a dynamic MER method and manual filtering to select songs with more emotional variety, in particular songs in the upper left and lower right quadrants of the VA space. This led to a different distribution of labels, which allowed to identify problems with valence recognition.

Estimating the absolute value of an emotion in real time could be difficult for the annotators, and often the direction of change is indicated correctly whereas the magnitude is not. We proposed to alleviate this problem by offsetting the annotations into the same bias using the overall emotion of the song (as annotated separately).

The valence–arousal model considered in the benchmark has been widely adopted in research on affective computing [2, 38, 71–73]. However, the model is not free of criticisms and some other alternatives may be considered in the future. For example, the VA model has been criticized for being too reductionist and that other dimensions such as dominance should be added [74]. Moreover, the terms ‘valence’ and ‘arousal’ may be sometimes too abstract for people to have a common understanding of its meaning. Such drawbacks of the VA model can further harm the inter-annotator agreement of the annotations for an annotation task which is already inherently fairly subjective.

In the benchmark, we resampled the annotations to either 1Hz or 2Hz. This led to benchmark participants using 1 or 0.5 second windows as the main unit of emotion prediction. As far as musical emotion is usually created on bigger time scales, the best algorithms for dynamic MER were those that could incorporate the bigger context, through either algorithm design (LSTM-RNN) or smoothing step applied at a later stage. Another way of performing dynamic MER is to first segment the emotional segments or use different units, such as scores, for emotion recognition [75].

The best feature-sets that were suggested for the task treated predicting valence and arousal separately, and suggested separate feature selection or dimensionality reduction steps for each emotional dimension. Again, it was shown that though arousal can be successfully modeled just with simple timbral features (spectral valley and spectral flatness), modeling valence is much more complex, and satisfactory performance was not achieved by any of the algorithms.

It is known that emotion perception is highly subjective, especially for valence [2]. Therefore, instead of taking the average values of the emotional annotations as the ground truth and training a generalized model for predicting them, we might want to have a look at the raw annotations and investigate the difference across the annotators. For example, it is possible that two songs with similar average ratings would have different variances in the raw annotation, and that it is better to explicitly model the variance computationally [27, 72]. It is also possible to build personalization techniques for customized MER predictions [76, 77], though to our best knowledge little has been done to personalize a dynamic MER model.

As shown in Table 1, the DEAM dataset contains rich extra meta-data about the songs and the annotators, such as the genre labels of the songs, the familiarity and liking of each of the annotated songs for each annotators, and even the personality traits of the annotators (in 2014). Such information can be studied and exploited in future work.

Although the benchmark is mainly designed for dynamic MER, the annotations, after being summarized over time, can also be useful for static MER. We also expect the dataset can facilitate the application and development of other mid- to high- level audio and non-audio features (e.g. [78, 79]), and other machine learning algorithms (e.g. that better account for temporal dynamics or personal differences) in the context of MER.

Emotion recognition from audiovisual signals is a task that is related to recognizing the spontaneous emotional expressions. Coutinho *et al.* [80] demonstrated that emotion recognition models can be transferred from speech to music and vice versa. As a result, there are parallels between the winning models in AVEC challenges that are addressing emotion recognition from human behavior and the ones addressing Emotion in Music task. In both cases, fine-tuned LSTM recurrent neural networks are the best performing models [60, 81].

## Conclusions

In this paper, we analyzed and summarized our findings in developing a new benchmark for emotional analysis in music. Analyzing three years of annotations on dynamic emotion recognition, we found them to be demanding in need of very conscientious and well trained annotators. We only succeeded in acquiring high quality labels on a crowdsourcing platform after directly engaging with workers and providing feedback in addition to a fair and mutually agreed compensation. We found that the results are less sensitive to the type of acoustic features, if we take enough of them into account. Recurrent neural networks and particularly LSTM is very effective in capturing the dynamic changes in emotion in music from acoustic features.

We release the data under Non Commercial Creative Commons (BY-NC) and we hope that this benchmark including its dataset and evaluation metrics helps accelerating research in MER.
